# Hippocampal connectivity in Amyotrophic Lateral Sclerosis (ALS): more than Papez circuit impairment

**DOI:** 10.1007/s11682-020-00408-1

**Published:** 2020-10-23

**Authors:** Francesca Trojsi, Federica Di Nardo, Giuseppina Caiazzo, Mattia Siciliano, Giulia D’Alvano, Teresa Ferrantino, Carla Passaniti, Dario Ricciardi, Sabrina Esposito, Luigi Lavorgna, Antonio Russo, Simona Bonavita, Mario Cirillo, Gabriella Santangelo, Fabrizio Esposito, Gioacchino Tedeschi

**Affiliations:** 1 Department of Advanced Medical and Surgical Sciences, MRI Research Center SUN-FISM, Università degli Studi della Campania “Luigi Vanvitelli”, Naples, Italy; 2 Department of Psychology, Università degli Studi della Campania “Luigi Vanvitelli”, Caserta, Italy; 3 Department of Medicine, Surgery and Dentistry, Scuola Medica Salernitana, University of Salerno, Baronissi, Salerno Italy

**Keywords:** Amyotrophic lateral sclerosis, Resting state functional MRI, Diffusion tensor imaging, Memory dysfunction, Papez circuit

## Abstract

Emerging evidence suggests that memory deficit in amyotrophic lateral sclerosis (ALS), a neurodegenerative disease with varying impairment of motor abilities and cognitive profile, may be independent from executive dysfunction. Our multimodal magnetic resonance imaging (MRI) approach, including resting state functional MRI (RS-fMRI), diffusion tensor imaging (DTI) and voxel-based morphometry (VBM), aimed to investigate structural and functional changes within and beyond the Papez circuit in non-demented ALS patients (n = 32) compared with healthy controls (HCs, n = 21), and whether these changes correlated with neuropsychological measures of verbal and non-verbal memory. We revealed a decreased functional connectivity between bilateral hippocampus, bilateral parahippocampal gyri and cerebellum in ALS patients compared with HCs. Between-group comparisons revealed white matter abnormalities in the genu and body of the corpus callosum and bilateral cortico-spinal tracts, superior longitudinal and uncinate fasciculi in ALS patients (p < .05, family-wise error corrected). Interestingly, changes of Digit Span forward performance were inversely related to RS-fMRI signal fluctuations in the cerebellum, while changes of both episodic and visual memory scores were inversely related to mean and radial diffusivity abnormalities in several WM fiber tracts, including middle cerebellar peduncles. Our findings revealed that ALS patients showed significant functional and structural connectivity changes across the regions comprising the Papez circuit, as well as more extended areas including cerebellum and frontal, temporal and parietal areas, supporting the theory of a multi-system pathology in ALS that spreads from cortical to subcortical structures.

**Electronic supplementary material:**

The online version of this article (10.1007/s11682-020-00408-1) contains supplementary material, which is available to authorized users.

## 1. Introduction

Amyotrophic lateral sclerosis (ALS), the most common motor neuron disease (MND), is a rapidly progressive neurodegenerative disorder primary involving upper and lower motor neurons (Hardiman et al. 2017). However, cognitive and behavioural deficits of varying severity are frequently reported in ALS (Beeldman et al. 2016). The recently revised Strong Criteria (Strong et al. 2017) further supported that behaviour and/or cognitive dysfunctions (i.e., executive and language impairments), not sufficient to diagnose frontotemporal dementia (FTD), could coexist with ALS (i.e., ALS with behavioural impairment [ALSbi], with cognitive impairment [ALSci], and with both [ALSbci]). Conversely, memory dysfunction, although extensively studied and observed in several cohorts of ALS patients (Mantovan et al. 2003; Machts et al. 2014; Abdulla et al. 2014; Christidi et al. 2017), when isolated, does not meet the criteria for diagnosis of ALSci.

The evidence of memory deficits in ALS, related to microstructural abnormalities, as revealed by neuropathologic (Nakano 1993; Takeda et al. 2009; Brettschneider et al. 2013) and neuroimaging (Abdulla et al. 2014; Stoppel et al. 2014; Christidi et al. 2014, 2017, 2019; Bueno et al. 2018) studies, drew attention to dysfunction of temporal lobe networks associated to an amnestic profile (Bede et al. 2013a; Machts et al. 2015). In particular, a specific focus of interest in histopathological studies was represented by the perforant pathway (PP) zone, which has been associated in ALS with detection of phosphorylated TAR DNA-binding protein 43 (pTDP-43) deposits (Geser et al. 2008; Brettschneider et al. 2013), as also confirmed *in vivo* by magnetic resonance imaging (MRI) data (Kassubek et al. 2014; Schmidt et al. 2016; Gorges et al. 2018). In this regard, advanced neuroimaging techniques, such as structural and functional MRI techniques, offered unprecedented opportunities to characterize hippocampal changes (Bede et al. 2013b, 2018; Abdulla et al. 2014; Stoppel et al. 2014; Westeneng et al. 2015; Machts et al. 2018; Christidi et al. 2018, 2019).

More recently, a multimodal MRI approach, combining cortical volume, voxel-based morphometry (VBM), diffusion tensor imaging (DTI) and resting state functional MRI (RS-fMRI) analyses, investigated the integrity of the “Papez circuit” (Brodal 2010) in a cohort of ALS patients compared to healthy controls (HCs) (Bueno et al. 2018), revealing that ALS patients not only showed significant structural changes, but also widespread functional connectivity abnormalities across regions comprising the Papez circuit.

On this background, in order to shed more light on the cerebral substrates of memory impairment in ALS within and also beyond some regions included in the Papez circuit aiming at replicating and increasing previous findings by Bueno et al. (2018), particularly by addressing more extensively the neuropsychological profile of the ALS population by ALS-specific and ALS non-specific tools, we performed a multimodal MRI study in a cohort of newly diagnosed, non-demented ALS patients and HCs. We investigated the structural integrity of gray and white matter (GM, WM) within and beyond several areas comprising the Papez circuit, by performing whole-brain VBM and DTI analyses and seed-based RS-fMRI analysis. Moreover, we explored the correlations between the obtained functional and structural connectivity measures and the most commonly used clinical measures of memory function.

## Methods

### Case selection

Thirty-two right-handed patients (25 M, 7 F; mean age 58.3 ± 10.3), with definite and clinical or laboratory-supported probable ALS, according to El-Escorial revised criteria (Brooks et al. 2000), showing a “classic” phenotype (Chiò et al. 2011), revealed to be able to tolerate the MRI exam, were consecutively recruited at the First Division of Neurology of the University of Campania “Luigi Vanvitelli” (Naples, Italy) from January 2018 to January 2019. Ten patients, unable to tolerate the MRI exam because of the physical disability in getting on to the scan table, were excluded.

Five patients had a bulbar onset and 27 a spinal onset. As for clinical features, we measured: disease duration (from symptom onset to scan date in months); ALSFRS-R total score (0–48, with lower total reflecting higher disability) and subscores (i.e., bulbar, fine-motor, gross-motor and respiratory subscores) (Cedarbaum et al. 1999); disease progression rate (48 minus current ALSFRS-R / disease duration); and upper motor neuron (UMN) score, index of pyramidal dysfunction through the evaluation of the number of pathologic reflexes elicited from 15 body sites (Turner et al. 2004).

Genetic analysis was performed in all patients, exploring *C9orf72* repeat expansion and mutations of *SOD1*, *TARDBP* and *FUS/TLS*. No mutations of these genes were reported.

Twenty-one right-handed HCs (15 M, 6 F; mean age 56 ± 10.1) were enrolled by “word of mouth” and among caregivers’ friends. They were age-, sex- and education-matched with the enrolled ALS patients. Moreover, they had no comorbid neurological, psychiatric or medical conditions. Exclusion criteria for all subjects were: medical illnesses or substance abuse that could interfere with cognitive functioning; any (other) major systemic, psychiatric, or neurological diseases; other causes of brain damage, including lacunae and extensive cerebrovascular disorders at MRI; a vital capacity lower than 70% of the predicted value (to prevent bias of respiratory compromise on cognitive measures).

### Neuropsychological assessment

To assess cognitive and behavioural profile of our population, neuropsychologists with specific expertise in ALS assessment (T.F., M.S., C.P.) administered a multi-domain battery to all participants. ALS patients and HCs underwent Mini- Mental State Examination (MMSE) (Folstein et al. 1975) and the Italian version of Edinburgh Cognitive and Behavioural ALS Screen (ECAS) (Poletti et al. 2016; Siciliano et al. 2017) assessing global cognitive functioning. ALS patients underwent: the digit span backward (Monaco et al. 2013), the Stroop test (Barbarotto et al. 1998), fluency with the phonemic (Carlesimo et al. 1996) and semantic (Spinnler and Tognoni 1987) fluency tests with the relative fluency indices (controlling for individual motor disabilities) (Abrahams et al. 2000) (executive functions); Digit Span forward and Corsi Block-Tapping test (Orsini et al. 1987) (verbal and visuo-spatial short-term memory); Rey Auditory Verbal Learning Test (RAVLT-immediate and delayed recall) (Carlesimo et al. 1996) (long term verbal memory); the Raven’s coloured progressive matrices (Carlesimo et al. 1996) (non-verbal intelligence); Hamilton depression rating scale (Hamilton 1960) (mood); and ALS-Frontotemporal Dementia-Questionnaire (ALS-FTD-Q) (Raaphorst et al. 2012) (behavioral disturbances) administered to patients’ caregivers.

For each subject, the scores obtained at each neuropsychological test was adjusted for age, education, and gender, as appropriate, and the performance was evaluated according to the percentile distribution in the corresponding normative populations. The 5th percentile was used as a cut-off.

### Statistical analysis: between-groups comparisons of clinical and neuropsychological data

Shapiro-Wilk tests were used to assess normality and, according to distribution of the data, t-test and Chi-square test were used to compare demographics and neuropsychological scores between ALS patients and HCs. The IBM SPSS v. 22.0 was used for all analyses and the level of significance was set at p < .05. For more details about correlation analyses between MRI and neuropsychological data, see the RS-fMRI and DTI sections.

### MRI analysis

#### Magnetic resonance imaging

Magnetic-resonance images were acquired on a 3T scanner equipped with an 8-channel parallel head coil (General Electric Healthcare, Milwaukee, Wisconsin). The imaging protocol included: three-dimensional T1-weighted images (gradient-echo sequence Inversion Recovery prepared Fast Spoiled Gradient Recalled-echo, time repetition = 6988 ms, TI = 1100 ms, TE = 3.9 ms, flip angle = 10, voxel size = 1 × 1 × 1.2 mm3); RS-fMRI sequence, consisting of 240 volumes of a repeated gradient-echo echo-planar imaging T2*-weighted sequence (time repetition = 1508 ms, axial slices = 29, matrix = 64 × 64, field of view = 256 mm, thickness = 4 mm, interslice gap = 0 mm, voxel size = 4 × 4 × 4 mm3); whole-brain DTI, performed using a GRE EPI sequence (repetition time = 10,000 ms, echo time = 88 ms, field of view = 320 mm, isotropic resolution = 2.5 mm, b value = 1,000 s/mm2, 32 isotropically distributed gradients, frequency encoding RL); T2-fluid attenuation inversion recovery to exclude severe cerebrovascular disease according to standard clinical neuroradiological criteria on visual inspection by three experienced radiologists. During the functional scan, subjects were asked to simply stay motionless, awake, and relax and to keep their eyes closed. The total scanning time for RS-fMRI was set to about 6 minutes, thereby the chance of falling asleep would be much reduced. Immediately after the scan, each participant was asked questions to verify their degree of cooperation. Moreover, all ALS patients had normal respiratory function that prevented them from suffering from specific (insomnia or hypersomina) sleep problems. No visual or auditory stimuli were presented at any time during functional scanning. The total duration of each scan was about 38 minutes.

#### RS-fMRI data preparation and preprocessing

Standard functional image data preparation and preprocessing, statistical analysis, and visualization were performed with the software BrainVoyager QX (Brain Innovation BV, Maastricht, The Netherlands). Data preprocessing included the correction for slice time acquisition, a three-dimensional rigid-body motion correction based on a 6-parameter rigid body alignment to correct for minor head movements, and the application of a temporal high pass filter with cut-off set to 3 cycles per time course (0.008 Hz). The scanner automatically exclude five scans at the beginning due to motion artefacts. Translational motion parameters were verified to be always less than 1 functional voxel for all included participants. Structural and functional data were coregistered and spatially normalized to the Talairach standard space using a 12-parameter affine transformation. During this procedure, the functional images were resampled to an isometric 3-mm grid covering the entire Talairach box.

#### Seed-based connectivity analysis

A seed-based analysis was performed to study functional connectivity from some Papez circuit areas, including bilateral hippocampus, bilateral parahippocampal gyrus (PHG), and anterior and posterior cingulate cortex (ACC and PCC), identified according to a previous analysis (Bueno et al. 2018), to the entire brain. For this purpose nuisance signals [global signal, WM and cerebrospinal fluid (CSF) signals] were regressed out from each data set together with motion translation and rotation estimates after segmenting the entire brain, the WM and ventricles from the normalized T1 volume.

**Fig. 1:**
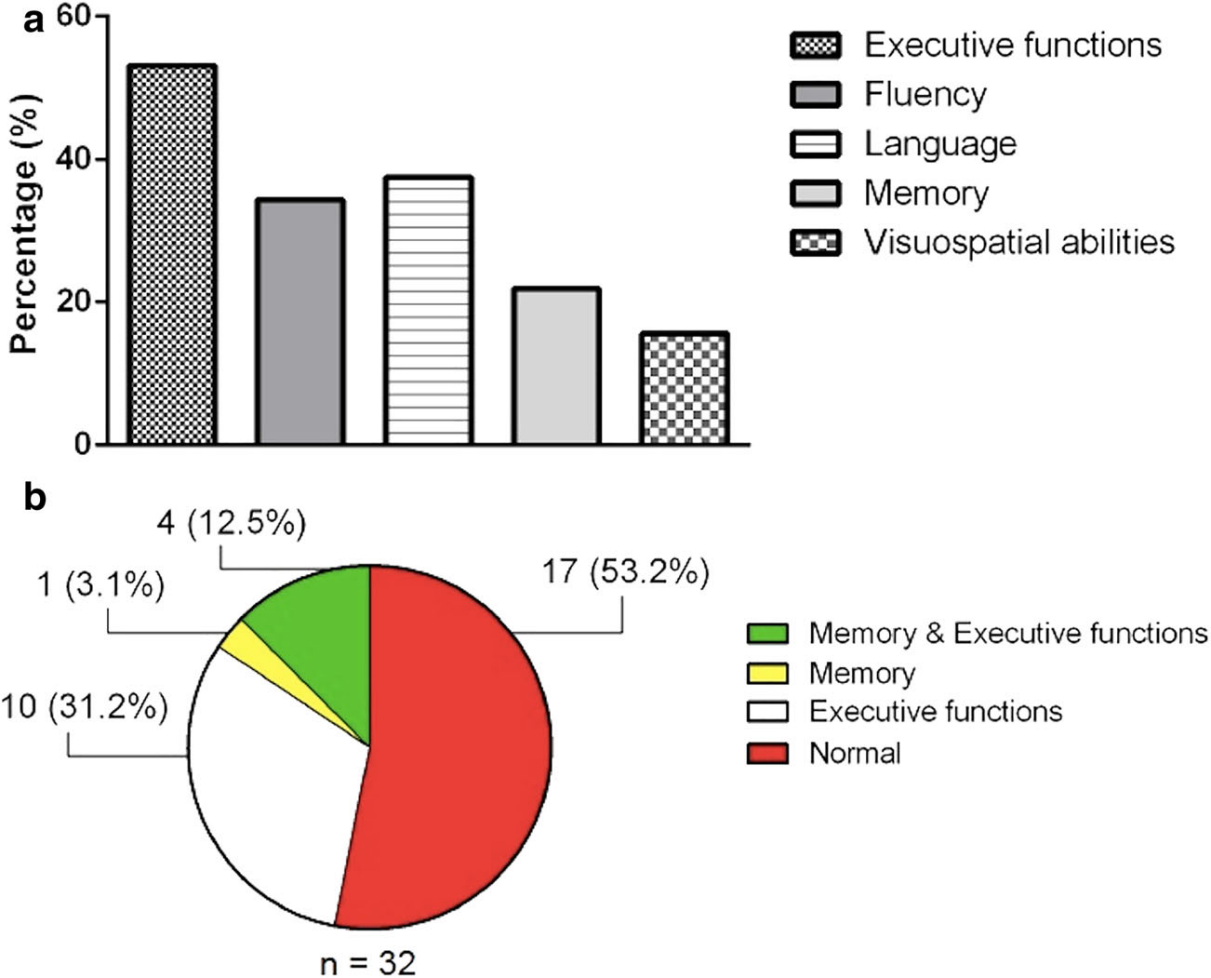
**A **Cognitive profile of the studied population Percentage of ALS patients falling below adjusted cut-offs across ECAS subdomains; **B** number (and percentage) of patients with abnormal performances in at least one neuropsychological test of memory, executive functions, both, or none

Six seed regions (right and left hippocampus; right and left PHG; ACC; PCC) were defined from anatomical brain atlas “Talairach-labels-1mm” in the Functional MRI of the Brain (FMRIB) Software Library (FSL) (Lancaster et al. 2007) (Supplementary materials - Fig. 1). To compute functional connectivity maps corresponding to a selected seed region of interest (ROI), the mean regional time course was extracted from all ROI voxels and correlated against all voxels of the brain. Separate correlation maps were produced for each subjects of each group and ROI. The correlation maps were applied the Fisher’s transform z = 0.5 Ln [(1 + r)/(1 – r)] before entering a second-level random-effects statistical analysis where the main and differential effects of the two studied groups were summarized as t-statistic maps. This analysis was carried out by treating the individual subject map values as random observations at each voxel, thereby the classical analysis of variance (ANOVA) was performed at each voxel to map the whole-brain distribution of the seed-based functional connectivity for the difference between the two groups, using age and gender as nuisance covariates. To correct for multiple comparisons in the voxel-based analysis, regional effects resulting from the voxel-based comparative tests were only accepted for compact cluster surviving the joint application of a voxel- and cluster-level threshold chosen with a nonparametric randomization approach. Namely, an initial voxel-level threshold was set to p = .001 (uncorrected) and a minimum cluster size was estimated after 1,000 Monte Carlo simulations that protected against false positive cluster up to 5% (Forman et al. 1995; Ecklund et al. 2016).

Individual z-scores from regions identified in the above analysis were also extracted and used in linear correlation analysis in ALS patients group with memory tests (i.e., RAVLT-immediate and -delayed recall, Digit Span forward and Corsi Block-Tapping tests) scores performed by “corrcoef” function of Matlab. For these regional analyses we used the Pearson linear correlation coefficient and a statistical significance level of p < .05 (Bonferroni corrected).

#### Diffusion tensor imaging (DTI) analysis

A whole-brain, voxel-based Tract-based spatial statistics (TBSS) approach was used for group analysis of DTI data (Smith et al. 2006). DTI data sets were processed with FSL software package v6.0 (www.fmrib.ox.ac.uk/fsl).

Preprocessing included eddy current and motion correction and brain-tissue extraction. After preprocessing, DTI images were averaged and concatenated into 33 (1 B = 0 + 32 B = 1000) volumes and a diffusion tensor model was fitted at each voxel, generating fractional anisotropy (FA), mean (MD) and axial diffusivity (AD) and eigenvalue (λ1, λ2, λ3) maps. The average of the second and third eigenvalues of the diffusion tensor was used for the definition of the radial diffusivity (RD). Images were warped to the Montreal Neurological Institute (MNI) 152 template, available as standard T1 data set in the FSL software package. TBSS was run with FA maps to create the “skeleton”, which represents the center of all fiber bundles in common to all subjects, and which was used for all other maps. To this purpose, FA images of all subjects (n = 53) were aligned to a common target (1 × 1 × 1 mm MNI152 FMRIB58_FA standard space) using nonlinear registration. A mean FA skeleton was then created with threshold of FA > 0.2. Individual skeleton images were submitted to a General Linear Model (GLM) analysis using randomize with appropriate design matrices and linear contrasts defined for the group comparisons and the correlations between all diffusivity parameters (FA, RD, AD, MD) and memory scores evaluated only in ALS patients (i.e., RAVLT-immediate and -delayed recall; Digit Span forward; Corsi Block-Tapping test). Age, gender and education were considered as covariates. The resulting statistical maps were thresholded at p < .05 (family-wise error [FWE] corrected) using the threshold-free cluster enhancement (TFCE) method (Smith et al. 2006). Moreover, the TBSS results were linked to standard anatomic data derived from the International Consortium of Brain Mapping DTI-81 WM (ICBM-DTI-81-WM) labels atlas (Johns Hopkins University, Baltimore, MD) (Wakana et al. 2007; Hua et al. 2008).

In addition to the whole-brain TBSS analysis, a volume of interest (VOI) analysis was also performed focusing on the 10 WM tracts most commonly found impaired in previous studies on ALS (Cirillo et al. 2012; Christidi et al. 2014, 2017; Agosta et al. 2016): body and genu of corpus callosum (CC); corticospinal tracts (CST); cingulum bundles; superior longitudinal fasciculus (SLF-Left/Right); and UF (Left/Right). Mean FA, MD, AD, and RD values within these tract labels in Montreal Neurological Institute (MNI) space were extracted from the spatially normalized and skeletonized FA, MD, AD, and RD maps of each individual. The DTI parameters of the VOIs were compared between the two groups of subjects using Mann-Whitney U test (p < .01, Bonferroni corrected).

#### Regional atrophy measurements: voxel-based morphometry (VBM)

We performed a whole-brain VBM analysis using the VBM toolbox (http://dbm.neuro.uni-jena.de/vbm.html) of SPM12 software package (http://www.fil.ion.ucl.ac.uk/spm/) with default parameters incorporating the DARTEL toolbox to obtain a high-dimensional normalization protocol (Ashburner 2007). Images were bias-corrected, tissue-classified, and registered using linear (12-parameter affine) and non-linear transformations (warping) within a unified model with default parameters incorporating the DARTEL toolbox. Subsequently, the warped GM segments were affine-transformed into MNI space and were scaled by the Jacobian determinants of the deformations to account for the local compression and stretching that occurs as a consequence of the warping and affine transformation (modulated GM volumes). Moreover, modulated images were smoothed with a 6-mm full-width half maximum Gaussian kernel to create the final probability maps (Henley et al. 2010). Harvard-Oxford cortical and subcortical structural atlases were used (Desikan et al. 2006). Unpaired t-test was used to compare ALS patients to HCs. GM atrophy results of between-group comparisons were FWE corrected at a level of p < .05 and covaried for age, gender and total intracranial volume (TIV; i.e., the sum of GM, WM and CSF volumes) (Malone et al. 2015).

## Results

### Demographics and neuropsychological variables

Patients and controls characteristics were reported in the Table 1. ALS patients and HCs did not statistically differ on age, gender and education. Considering that most subjects were men (about 70%), to minimize possible influence of gender in the results, statistical analyses were implemented considering also gender as a covariate. Two-tailed t-test revealed no significant difference in global cognitive performances (i.e., Mini-Mental State Examination, MMSE; total score of Edinburgh Cognitive and Behavioural ALS Screen, ECAS) between ALS patients and HCs. However, in our sample the executive function, fluency, and language subscores were more impaired in comparison to memory or visuospatial ability subscores (Table 1; Fig. 1A). On the base of ECAS subscores (Poletti et al. 2016; Siciliano et al. 2017), according to the Strong criteria for frontotemporal spectrum disorder of ALS (2017), 6 patients had ALSci (i.e., 5 with executive functions impairment and 1 with both executive and language impairments), 12 patients had ALSbi, and 2 patients had ALSbci. Finally, when evaluating the performances at the neuropsychological tests specifically assessing executive functions and memory cognitive domains, we revealed that deficits of memory and executive function coexisted in 12,5% of the studied patients. Moreover, about the half of the patients did not show executive or memory deficits (Fig. 1B).

**Table 1:** Demographic, clinical, and neuropsychological measures of patients and healthy controls; data are shown as mean (standard deviation) or count (percentage)

Variable	ALS patients (n = 32)	HCs (n = 21)	*t-*test/χ^2^	*p*-value	Below\above cut-off score^a^
Demographics:					
Age, years	58.3 (10.3)	56.0 (10.1)	− 0.8	0.40	-
Education, years	10.5 (4.3)	12.5 (3.04)	1.8	0.10	-
Sex, male	25 (78%)	15 (71%)	0.3	0.60	-
Clinical features:					
Symptom duration, months	19.5 (24.6)	-	-	-	-
ALSFRS-R total score	39.0 (6.8)	-	-	-	-
Bulbar subscore	10.7 (2.2)	-	-	-	-
Fine motor subscore	8.5 (3.0)	-	-	-	-
Gross motor subscore	8.6 (2.9)	-	-	-	-
Respiratory subscore	11.1 (1.9)	-	-	-	-
Disease Progression Rate*	0.8 (0.7)	-	-	-	-
Upper Motor Neuron score	7.1 (5.0)	-	-	-	-
Neuropsychological measures:					
Global cognition:					
Mini Mental State Examination	28.3 (1.6)	28.5 (1.1)	0.5	0.60	0
ECAS total score	90.9 (21.3)	100.6 (9.0)	1.9	0.10	4
ECAS subscores:					
Executive functions	29.18 (9.75)	34.29 (5.35)	2.1	0.03	17
Fluency	16.22 (7.26)	17.67 (4.64)	0.7	0.43	11
Language	21.68 (4.04)	22.05 (2.69)	0.3	0.71	12
Memory	13.04 (5.35)	15.86 (3.21)	2.1	0.03	7
Visuospatial abilities	10.7 (1.6)	10.7 (1.3)	0.1	0.91	5
Executive functions:					
Letter fluency task	26.9 (11.8)	-	-	-	5
Semantic fluency task	20.1 (6.0)	-	-	-	1
SF Index**	29.2 (13.4)	-	-	-	-
PF Index**	9.0 (11.3)	-	-	-	-
Stroop test – seconds	24.6 (10.0)	-	-	-	3
Stroop test – number of errors	1.0 (2.5)	-	-	-	1
Memory:					
RAVLT-immediate recall	38.5 (9.0)	-	-	-	3
RAVLT-delayed recall	8.2 (2.4)	-	-	-	3
Digit Span forward	5.1 (0.9)	-	-	-	1
Digit Span backward	3.2 (0.8)	-	-	-	9
Corsi Block-Tapping test	4.8 (0.7)	-	-	-	0
Non verbal Intelligence:					
Coloured Progressive Matrices	26.3 (4.9)	-	-	-	0
Behavioural assessment:					
ALS-FTD Q (cut-off ≥ 22)	13.1 (9.7)	-	-	-	4
Hamilton HDRS	10.0 (4.2)	-	-	-	2

Note. ALS, Amyotrophic Lateral Sclerosis; ALSFRS-R, ALS Functional Rating Scale Revised; ALS-FTD-Q, ALS-Frontotemporal Dementia questionnaire; ECAS, Edinburgh Cognitive and Behavioural ALS Screen; HCs, Healthy Controls; PF, Phonemic fluency; RAVLT, Rey’s Auditory Verbal Learning Test; SF, Semantic fluency; χ2, Chi-square test. *Disease Progression Rate was computed as: 48 – ALSFRS-R/Disease Duration. **Verbal fluency indices were obtained as following: time for generation condition - time for control condition (reading or writing generated words)/total number of items generated; ^**a**^ number of patients with cognitive performance\behavioural symptoms below\above cut-off score

### RSN functional connectivity: seed-based analysis

**Fig. 2:**
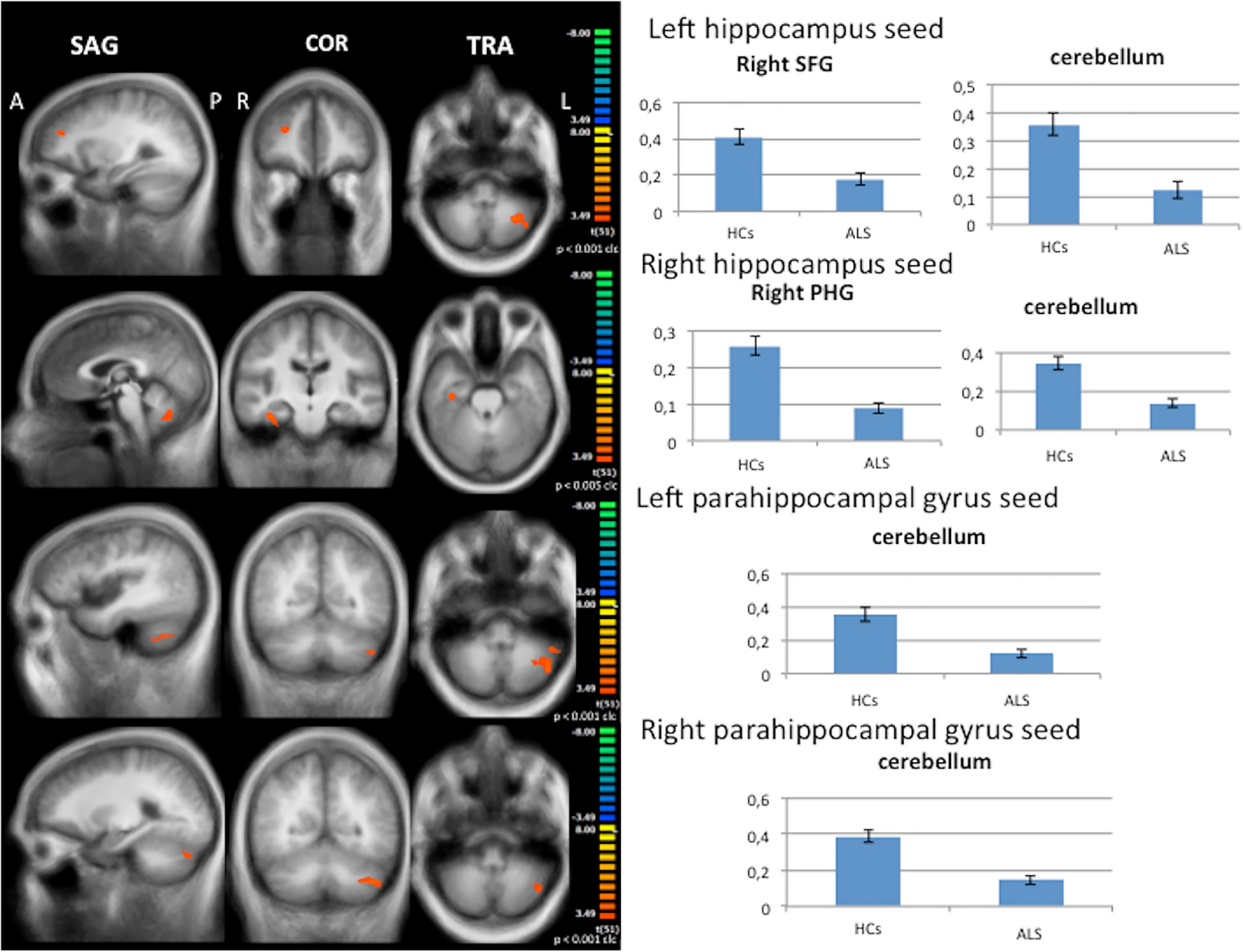
Seed-based connectivity analysis. Decreased functional connectivity between left and right hippocampus and left and right PHG, as seeds, and other brain areas within and beyond Papez circuit in ALS patients compared to HCs (left panels: between-group comparisons maps, red-yellow scale; right panels: bar plots of the average functional connectivity levels). A = anterior; clc = cluster-level corrected; COR = coronal; L = left; P = posterior; PHG = parahippocampal gyrus; R = right; SAG = sagittal; SFG = superior frontal gyrus; TRA = transverse

When compared to HCs, considering the left hippocampus as seed, decreased functional connectivity was revealed between the left hippocampus and the right superior frontal gyrus and the left lobule VIIIA of cerebellum (*p* < .001 cluster-level corrected) in the ALS group (Fig. 2). Decreased functional connectivity was found between the right hippocampus and the right posterior PHG and the left hemisphere of cerebellum (crus I) (*p* < .005 cluster-level corrected) in the ALS group (Fig. 2). When the right and the left posterior PHG were the seeds, decreased functional connectivity was observed between these seeds and cerebellum (left crus I) (*p* < .001 cluster-level corrected) in the ALS group (Fig. 2).

**Fig. 3:**
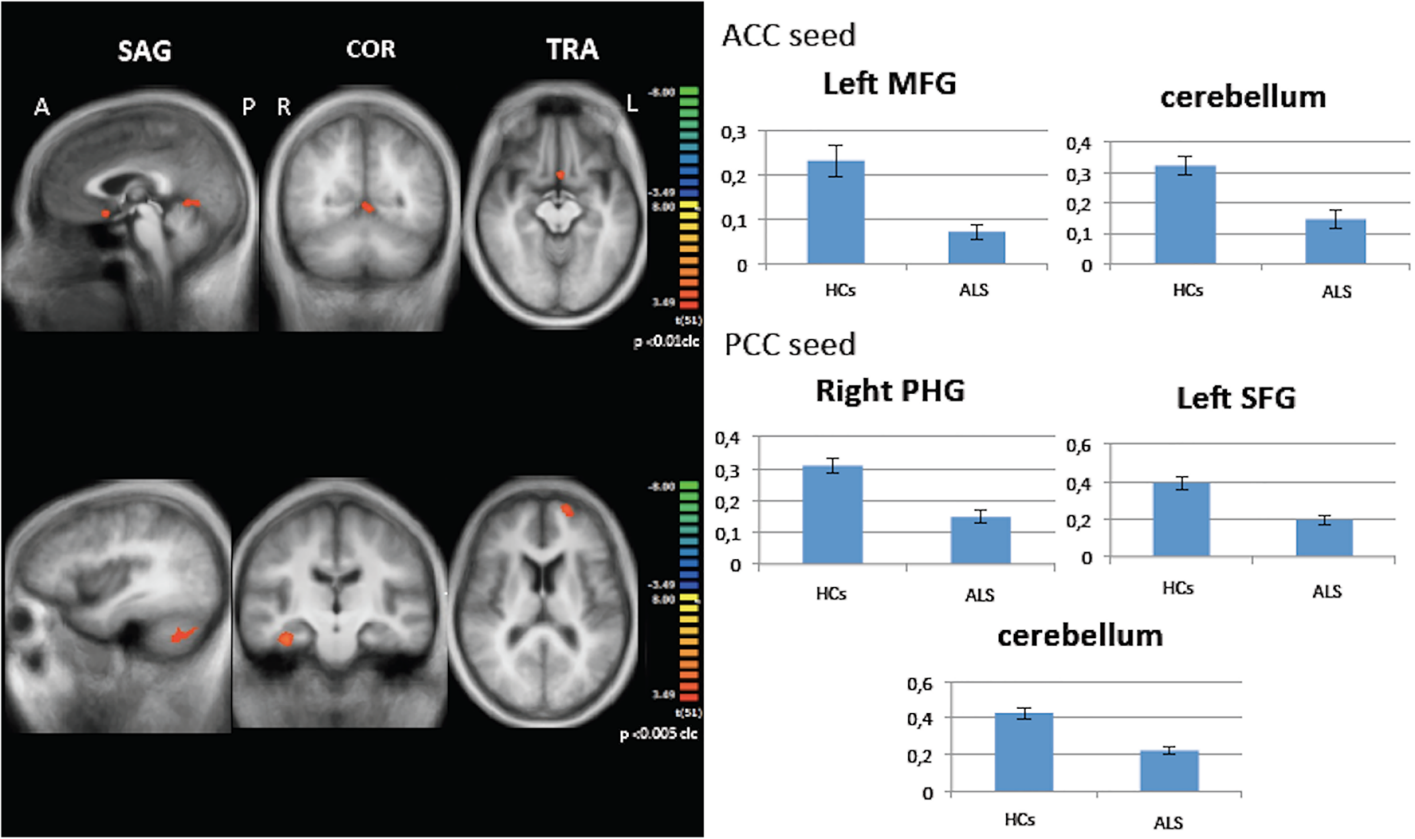
Seed-based connectivity analysis. Decreased functional connectivity between ACC and PCC, as seeds, and other brain areas within and beyond Papez circuit in ALS patients compared to HCs (left panels: between-group comparisons maps, red-yellow scale; right panels: bar plots of the average functional connectivity levels). A = anterior; ACC = anterior cingulate cortex; clc = cluster-level corrected; COR = coronal; L = left; MFG = middle frontal gyrus; P = posterior; PCC = posterior cingulate cortex; R = right; SAG agittal; SFG = superior frontal gyrus; TRA = transverse

Considering ACC as seed, decreased functional connectivity was found in ALS patients compared with HC between left middle frontal gyrus and the left lobule V of the cerebellum (p < .01 cluster-level corrected) in the ALS group (Fig. 3).

PCC showed decreased functional connectivity with the right posterior PHG, the left superior frontal gyrus and cerebellum (left crus I) (*p* < .005 cluster-level corrected) in the ALS group (Fig. 3).

No significant correlation was reported between memory scores and RS-fMRI connectivity measures (mean z-scores in the areas of altered FC) after correction for multiple comparisons.

### TBSS DTI analysis

#### Between-group analysis

**Fig. 4:**
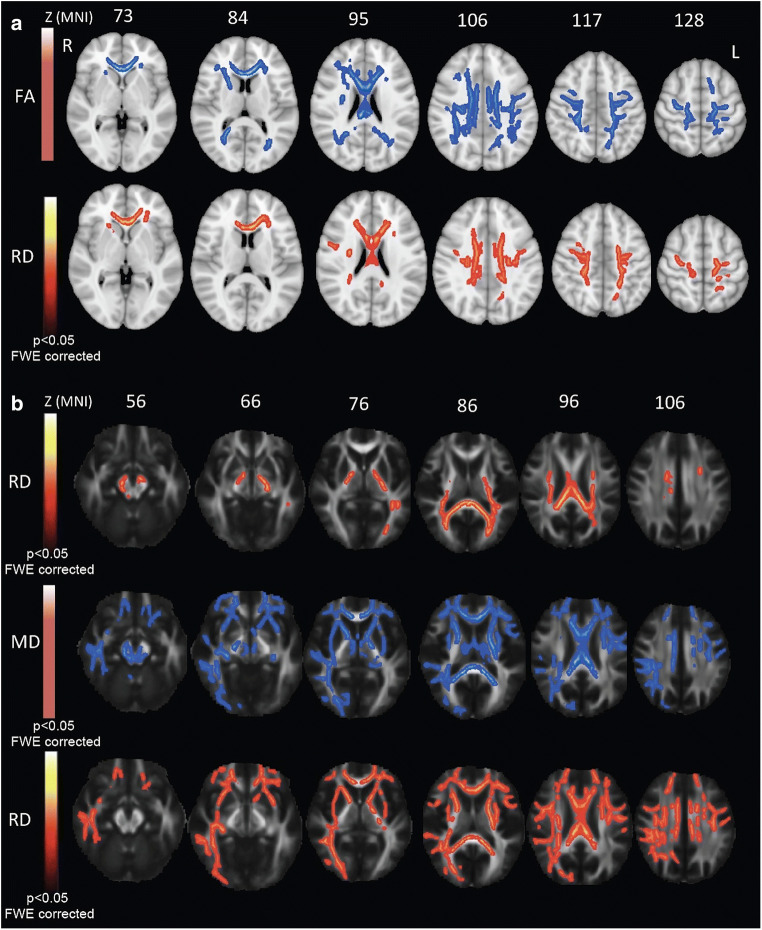
**A** TBSS DTI analysis. Comparison between FA (blue; upper panel) and RD (red-yellow; lower panel) statistic parametric maps of ALS patients versus HCs (*p* < .05, FWE corrected). **B** Voxel-wise correlation analysis between RD metrics and RAVLT immediate recall scores (red-yellow scale; upper panel); between MD metrics and Corsi Block-Tapping test score (blue scale; middle panel); and between RD metrics and Corsi Block-Tapping test score (red-yellow scale; lower panel) (*p* < .05, FWE corrected). FA = fractional anisotropy; MD = mean diffusivity; MNI = Montreal Neurological Institute; L = left; R = right; RAVLT = Rey Auditory Verbal Learning Test; RD = radial diffusivity

When compared to HCs, ALS patients exhibited decreased FA (*p* < .05, family-wise error [FWE] corrected) in the body and genu of CC, in the right and left CSTs underneath precentral gyri, in the right and left UF and in the right and left SLF (Fig. 4A), as also confirmed by VOI-based analysis (*p* < .01, Bonferroni corrected) (Supplementary materials, Table 2). Between-group comparisons revealed also significant increased RD measures in the same brain areas (Fig. 4A, Supplementary materials Table 2).

Between-group comparison revealed no significant differences in AD and MD measures.

#### Voxel-wise correlation analysis

We detected significant negative correlations (Fig. 4B): between RAVLT immediate recall scores and RD measures in the body and splenium of CC, in bilateral CSTs, in the left ILF and in the middle cerebellar peduncles (*p* < .05, FWE corrected); between Corsi Block-Tapping test and MD measures in the body and splenium of CC, in bilateral CSTs, in bilateral UF and SLF, in the right cingulum bundle and in the right ILF (*p* < .05, FWE corrected); between Corsi Block-Tapping test and RD measures in the body and splenium of CC, in bilateral CSTs, UF, SLF, ILF and cingulum bundles (*p* < .05, FWE corrected).

### VBM analysis

No significant difference was revealed in GM comparing ALS patients and HCs (*p* < .05, FWE corrected).

## Discussion

In this study, using a multimodal MRI approach, we revealed RS-fMRI changes in several brain areas within and beyond the Papez circuit in a sample of non-demented ALS patients, compared to HCs. Our analysis recalled the previous multimodal investigation by Bueno et al. (2018) focusing on Papez circuit functional and structural abnormalities in non-demented ALS patients compared to HCs. Moreover, structural (i.e., VBM, DTI) and functional (i.e., RS-fMRI) data were associated with changes of episodic memory performance, as assessed by memory subscores of the Addenbrooke’s Cognitive Examination-Revised (ACE-R) (Mioshi et al. 2006). In comparison to this study, although performing a similar multimodal MRI approach, we used both ALS-specific (i.e., ECAS) and not ALS-specific neuropsychological tests, for assessing global cognitive functions, and a list learning test (i.e., RAVLT), for assessing hippocampus-mediated verbal episodic memory, also previously used in ALS (Christidi et al. 2012). In our patients sample, only few ALS patients scored below the cut-offs for memory subdomain of ECAS, RAVLT-immediate and delayed recall and Digit Span forward tests, showing a major percentage of patients falling below cut-offs across executive, language and fluency domains, in line with previous evidence (Abrahams et al. 2014; Poletti et al. 2016), and a coexistence of executive and memory dysfunctions in 12,5% of the group (Fig. 1). We did not perform a *post-hoc* analysis by comparing memory impaired versus memory-unimpaired subsets of patients, because of the small subsets of patients. However, remarkably, a prospective multimodal MRI study by Christidi et al. (2019) revealed a disease-specific vulnerability profile of mesial temporal lobe structures in ALS patients in comparison to Alzheimer’s disease patients and HCs, defining the involvement of interconnected structures also in patients with different memory performances by a connectivity-based approach. To note, behavioural impairment, detected in 43% of the studied population, may recall the disease continuum existing between ALS and the behavioural variant of FTD (bvFTD), also highlighted by previous evidence of some overlap of brain functional connectivity abnormalities by comparing ALS to bvFTD patients (Trojsi et al. 2015), although different patterns of GM atrophy have been described in cohorts of patients affected by the two syndromes (Bueno et al. 2020).

Unexpectedly, we detected decreased functional connectivity between all the studied seeds and the left hemisphere of the cerebellum. The abnormal connectivity between the studied Papez circuit areas and the left hemisphere of the cerebellum, particularly the left crus I, represents the novel finding of our analysis, that reconciles with the increasing evidence of the extended role of the cerebellum in cognition beyond motor control (Habas et al. 2009; Shiroma et al. 2016). Firstly, Habas et al. (2009) revealed that distinct neocerebellar regions were involved in distinct cognitive functions. In particular, most of the neocerebellum (i.e., crus I-II) was shown to selectively contribute to cortico-cerebellar loops involved in executive control and episodic memory. More recently, Shiroma et al. (2016), using fMRI to investigate subjects with cerebellar tumors performing an established lure task, revealed that tumors compressing the posterior lateral cerebellum might affect the ability to separate patterns and to discriminate between similar experiences, on the basis of episodic memory. Along this line, Paleja et al. (2014) had previously investigated the functional networks underlying pattern separation tasks, underlining the role of a (secondary) cerebellar-hippocampal network.

Our evidence of impaired functional connectivity between Papez circuit areas and left middle and superior frontal regions contributes to underline the interaction between the frontal system and the temporal hippocampal system (Kramer et al. 2005). Specifically, the frontal system, underpinning executive functions, already shown to be mostly impaired in the ALS cognitive profile (Beeldman al. 2016; Consonni et al. 2016) like in our cohort of patients, has been also revealed to play the role of controlling and organizing the encoding and retrieval of episodic and visual memory information via the selection and implementation of relevant strategies (Kramer et al. 2005; Isingrini and Taconnat 2008; Burke et al. 2017). Furthermore, in agreement with the interaction between the frontal and the temporal hippocampal systems, episodic memory deficits are also likely to reflect the degradation of frontal abilities (Glisky 2007; Wascher et al. 2012).

As for results from whole-brain VBM and DTI analyses, the evidence of no significant cortical atrophy and of FA decrease and RD increase in the body and genu of CC, in the CSTs underneath precentral gyri and in bilateral UF and SLF in the ALS group compared to HCs recalls previous findings from the between-group analyses of different cohorts of patients (Cirillo et al. 2012; Raaphorst et al. 2015; Agosta et al. 2016). Of note, UF microstructural changes have been related to verbal episodic memory in ALS (Christidi et al. 2014), although in our study RAVLT immediate recall scores were found inversely related to RD measures in the left ILF and in the middle cerebellar peduncles, among non-pyramidal WM tracts. Beyond the previously commented involvement of cerebellum in episodic memory circuit, WM abnormalities in the right ILF have been recently related to episodic memory dysfunction in a population of subjects exhibiting brain WM hyperintensity with or without mild cognitive impairment (MCI) (Chen et al. 2019). In this study, MD increase in the right ILF and in the right inferior fronto-occipital fasciculus was shown to significantly contribute to differentiate MCI from non-MCI subjects. However, in our between-group analysis MD values were not found increased in ALS patients, probably due to a “pseudo-normalization” of the cellular density in the damaged areas as a consequence of reactive gliosis (Cirillo et al. 2012). Furthermore, the findings by Lockhart et al. (2012), who described widespread WM abnormalities, related to episodic memory impairment, in frontal, parietal, and subcortical regions, resemble the pattern of significant inverse correlation between Corsi Block-Tapping test score and MD and RD measures in extended frontotemporal (e.g., UF), frontoparietal (e.g., SLF) and temporoparietal (e.g., ILF) WM tracts identified in our study. Altogether these results strongly suggest that memory performance, in non-demented ALS patients as well as in older adults, is particularly vulnerable to injury in the connections between the frontal, temporal, and parietal cortex and their targets.

Strengths of our study are the combination of neuropsychological tests and MRI data, the comparison to a matched control group, and the use of normative scores to calculate individual performances. However, there are some shortcomings to be acknowledged, including some methodological issues, such as the cross-sectional design; the lack of ROI analysis of the VBM data, of complementary structural analyses (e.g., hippocampal segmentation, vertex analyses, and WM tractography) focused on the Papez circuit and of functional connectivity analysis encompassing all the areas comprising the Papez circuit; the relatively small sample size; the absence of correction for motor disability for some tests (e.g., Corsi Block tapping-test); the unavailability of the same cognitive measures for HCs; and the global signal regression approach used in the preprocessing of RS-fMRI data. Moreover, a substantial limit of all MRI studies is the selection bias of excluding ALS patients in advanced stages of disease (Turner and Modo 2010).

## Conclusions

Functional and structural connectivity abnormalities of the Papez circuit and beyond in ALS may suggest an extra-motor impairment of a cortico-cerebellar loop underpinning memory changes, although GM abnormalities are still unapparent. Moreover, our findings support an expanded role of the cerebellum beyond motor control and reinforce the concept of a multi-system degeneration in ALS, according to a spreading pattern of disease from cortical areas to subcortical structures, including precerebellar nuclei, via axonal transport (Brettschneider et al. 2013). Future longitudinal studies, performed in larger samples, will be needed to refine our results and better describe the extent and nature of impairment of brain circuits underpinning memory performance in ALS.

## Electronic Supplementary Material

 ESM 1 (DOCX 265 KB)

ESM 2 (DOCX 29.8 KB)
