# Supplementary material for: Hippocampal connectivity in Amyotrophic Lateral Sclerosis (ALS): more than Papez circuit impairment
Source: Brain Imaging Behav. 2020 Oct 23;15(4):2126–38. doi: 10.1007/s11682-020-00408-1 (PMC8413176; doi:10.1007/s11682-020-00408-1)
Supplement: Supplementary file 1 — (DOCX 265 KB) [file 11682_2020_408_MOESM1_ESM.docx]

**Supplementary materials**

**Fig. 1 Seed regions from anatomical brain atlas “Talairach-labels-1mm” in FSL** (Lancaster et al., 2007): Right (pink) and left hippocampus (yellow); right (violet) and left PHG (light green); ACC (red); PCC (green). A = anterior; ACC = anterior cingulate cortex; COR = coronal; L = left; P = posterior; PCC = posterior cingulate cortex; PHG = parahippocampal gyrus; R = right; SAG = sagittal; TRA = transverse.

**
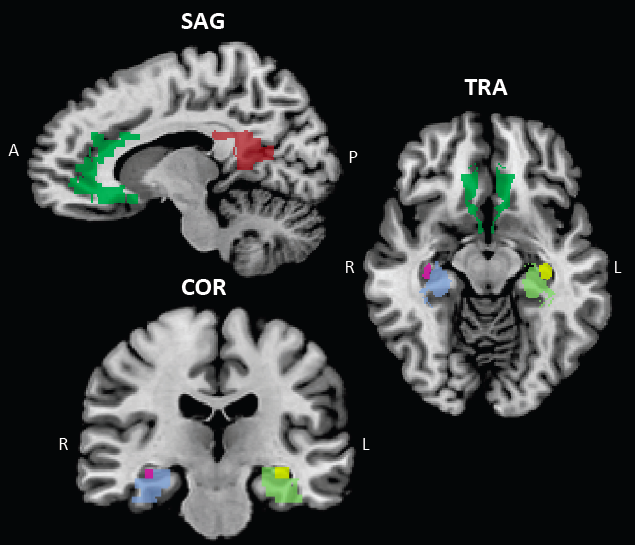
**
