# Supplementary material for: Hippocampal connectivity in Amyotrophic Lateral Sclerosis (ALS): more than Papez circuit impairment
Source: Brain Imaging Behav. 2020 Oct 23;15(4):2126–38. doi: 10.1007/s11682-020-00408-1 (PMC8413176; doi:10.1007/s11682-020-00408-1)
Supplement: Supplementary file 2 — (DOCX 29.8 KB) [file 11682_2020_408_MOESM2_ESM.docx]

| DTI parameters | VOIs  (MNI x,y,z) | mean | HCs  (confidence interval) | mean | ALS  (confidence interval) |
| --- | --- | --- | --- | --- | --- |
| FA | CC body  0; -9; 28 | 6,31E-01 | 6,20E-04 | 5,66E-01 | 5,33E-04 |
|  |  |  |  |  |  |
|  |  |  |  |  |  |
|  | CST-R  22; -33; 35 | 4,71E-01 | 3,62E-04 | 4,37E-01 | 4,09E-04 |
|  |  |  |  |  |  |
|  | CST-L  -22; -33; 35 | 4,69E-01 | 4,43E-04 | 4,30E-01 | 1,28E-04 |
|  |  |  |  |  |  |
|  | cingulum bundle-R  9; 17; 27 | 4,74E-01 | 4,09E-04 | 4,44E-01 | 3,79E-04 |
|  |  |  |  |  |  |
|  | cingulum bundle-L  -9; 17; 27 | 5,12E-01 | 4,77E-04 | 4,79E-01 | 3,69E-04 |
|  |  |  |  |  |  |
|  | UF-R  27; 13; -7 | 4,88E-01 | 4,57E-04 | 4,61E-01 | 4,05E-04 |
|  |  |  |  |  |  |
|  | UF-L  -27; 13; -7 | 4,19E-01 | 5,71E-04 | 3,97E-01 | 3,57E-04 |
|  |  |  |  |  |  |
|  | SLF-R  39; -14; 30 | 4,31E-01 | 3,77E-04 | 4,01E-01 | 3,45E-04 |
|  |  |  |  |  |  |
|  | SLF-L  -39; -14; 30 | 4,46E-01 | 4,20E-04 | 4,14E-01 | 3,31E-04 |
|  |  |  |  |  |  |
| RD | CC body  0; -9; 28 | 4,56E-04 | 9,41E-07 | 6,02E-04 | 7,45E-07 |
|  |  |  |  |  |  |
|  |  |  |  |  |  |
|  | CST-R  22; -33; 35 | 5,29E-04 | 3,52E-07 | 5,64E-04 | 4,24E-07 |
|  |  |  |  |  |  |
|  | CST-L  -22; -33; 35 | 5,07E-04 | 4,43E-07 | 5,43E-04 | 4,19E-07 |
|  |  |  |  |  |  |
|  | UF-R  27; 13; -7 | 6,11E-04 | 5,71E-07 | 6,43E-04 | 4,26E-07 |
|  |  |  |  |  |  |
|  | UF-L  -27; 13; -7 | 6,23E-04 | 6,88E-07 | 6,51E-04 | 4,03E-07 |
|  |  |  |  |  |  |
|  | SLF-R  39; -14; 30 | 5,61E-04 | 3,73E-07 | 5,90E-04 | 5,60E-04 |
|  |  |  |  |  |  |
|  | SLF-L  -39; -14; 30 | 5,29E-04 | 4,21E-07 | 3,76E-07 | 3,31E-07 |
|  |  |  |  |  |  |

**Table 2** Confidence intervals of FA and RD in the volumes of interest (VOIs), derived from Johns Hopkins University (JHU, Baltimore, Maryland) white matter tractography Atlas of FSL (Wakana et al., 2007; Hua et al., 2008), in which p<.01 (Bonferroni corrected) by comparing patients to healthy controls.

Note. ALS, amyotrophic lateral sclerosis; CC, corpus callosum; CST, cortico-spinal tract; DTI, diffusion tensor imaging; FA, fractional anisotropy; FSL, Functional MRI of the Brain (FMRIB) Software Library; HCs, healthy controls; L, left; MNI, Montreal Neurological Institute; R, right; RD, radial diffusivity; SLF, superior longitudinal fasciculus; UF, uncinate fasciculus; VOI, volume of interest.
